# Endoscopic Ear Surgery: Minimally Invasive, Maximum Views

**DOI:** 10.3390/jcm15041369

**Published:** 2026-02-09

**Authors:** Gerard P. Sexton, Ivan J. Keogh

**Affiliations:** 1 Academic Department of Otorhinolaryngology, University of Galway, H91 TK33 Galway, Ireland; 2 Department of Otorhinolaryngology, Head and Neck Surgery, University Hospital Galway, H91 YR71 Galway, Ireland

**Keywords:** endoscopic ear surgery, otology, minimally invasive ear surgery, endoscopic tympanoplasty, middle ear

## Abstract

Ear surgery has historically been performed with a microscope, an approach which often requires a post-auricular incision and represents a relatively invasive form of access to the middle ear. Over the past three decades, the advent of endoscopic techniques has allowed for a minimally invasive alternative. Endoscopic ear surgery (EES) has emerged as an innovative approach in otology, transforming the way surgeons address ear disorders and improving patient outcomes. EES provides enhanced visualization and exceptional precision during otologic procedures. This article examines the technical aspects of EES, its impact on contemporary otology practice, and the future direction of this technology.

## 1. Introduction

Endoscopic ear surgery (EES) represents the single most significant area of innovation and development in the recent history of otologic surgery [1]. As an alternative to microscopic ear surgery (MES), itself a time-honoured but relatively more invasive approach, there is a growing body of evidence in support of the routine use of EES across a broad array of otologic pathology [2,3,4]. The advocacy of dedicated pioneers worldwide and the incremental development of bespoke equipment by industry have transformed EES from an experimental and primarily diagnostic tool into a recognized therapeutic modality [5,6,7].

This article explores the history, principles, and technical aspects of EES, its advantages over traditional methods, the scope of its application, and its future within otology.

## 2. Anatomy and History of Ear Surgery

Before exploring EES, it is essential to understand the historical evolution of otologic surgery. The middle ear is anatomically complex, with close relations to critical structures including the brain, facial nerve, and major blood vessels. The tight confines of the external auditory canal and the middle ear cavity made mere visualization of the middle ear challenging for the greater part of human history. The first combined aural and nasal speculum was illustrated in 1363, and early otoscopes continued to rely on reflected sunlight until 1730 [8,9]. Even with the benefit of cadaveric models, much of the physiology of the ear remained poorly understood until the advent of microscopy, with obvious ramifications for the prognosis of otologic pathology.

With the development and rollout of the first widely available binocular surgical microscopes in 1951, otologic surgery underwent a paradigm shift [10]. The microscope provided both magnification and illumination, enabling surgeons to operate on delicate structures, such as the ossicular chain and the tympanic membrane, with greater precision and accuracy. MES became the standard for otologic procedures and gave acceptable exposure to facilitate tympanoplasty and stapedectomy, thereby widening the spectrum of diseases that could be addressed surgically. Modified radical mastoidectomy was also developed as an alternative to the now largely historical radical mastoidectomy.

Despite the significant advantages offered by MES, there remained several limitations. The depth of the operative field and the degree of magnification necessitated a parallax-free light source aligned with the operator’s field of view [11]. Though this is a feature of binocular operating microscopes, the natural curvature of the human ear canal often necessitated a postauricular incision to expose the middle ear to the light source adequately. Additionally, the potential for recidivism within the recesses of the middle ear required either escalation of surgery or a better means of assessing these areas.

## 3. Principles of Endoscopic Ear Surgery

**Figure 1:**
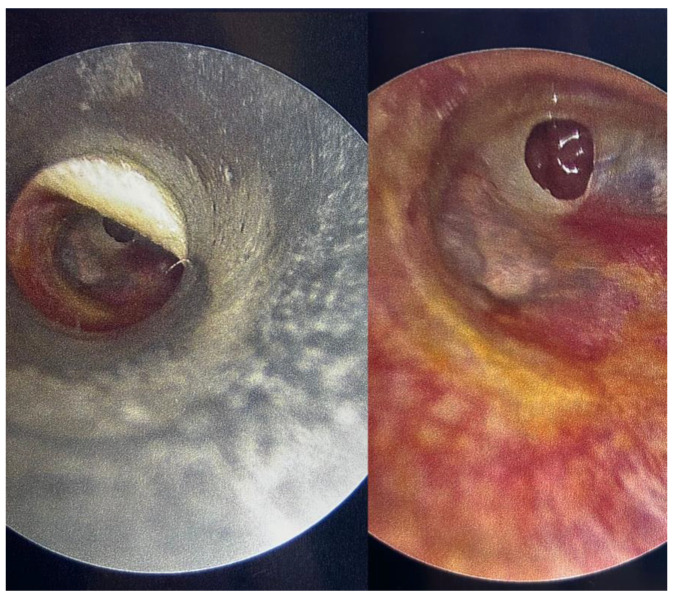
Microscopic (**left**) versus endoscopic (**right**) view of the same tympanic membrane with an anterior tympanic membrane perforation.

In the 1990s, endoscopic techniques were introduced into otologic surgery. While the field of view during MES is determined by the narrowest point of the ear canal, the basic principle of EES is that the endoscope bypasses any narrow areas and facilitates a wide and magnified field of view. First derived from experiences in rhinology, the endoscope was initially used only as an adjunct to MES for the evaluation of areas such as the sinus tympani [5]. This was followed shortly by the use of the endoscope as an operating tool in place of the microscope [12]. Although initially met with scepticism, the advantages of EES in terms of visualization and access rapidly became apparent. This was bolstered by the success rates reported by early practitioners, which have been borne out in many institutions worldwide [13,14,15]. Figure 1 illustrates the dramatic difference in the field of view achievable with a speculum and operating microscope when compared to an endoscope.

Many surgeons continue to prefer the microscope for its versatility and ability to facilitate two-handed surgery. Even for those not conversant in EES, the endoscope is increasingly recognized as a useful adjuvant for decision making [16]. In recognition of the varying levels to which the endoscope can be employed intraoperatively, Cohen et al. developed a classification of the degree to which the endoscope is used for documentation purposes [17].

One-handed technique is another key element that further distinguishes EES from MES. The traditional setup for MES takes advantage of both available hands for bimanual manipulation or the concurrent use of suction/irrigation systems. In EES, one hand (typically the non-dominant hand) holds the endoscope, while the dominant hand is used for surgical instruments. While sometimes considered a limitation of EES, this feature encourages the use of precise instrumentation as facilitated by improved access and visualization. Two-handed techniques are also described where an endoscope holder is used to mount the endoscope in the desired position but are less widely used [18,19].

## 4. Key Equipment Used in EES

**Figure 2:**
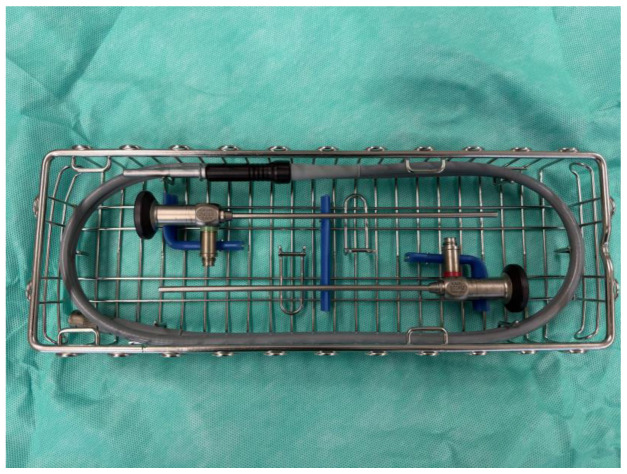
0-degree (upper) and 30-degree (lower) rigid endoscopes with light lead.

EES involves the use of a rigid endoscope that is inserted through the external auditory canal to provide a magnified, high-definition view of the middle and inner ear structures. The 14 cm long endoscope is the most widely used. Endoscopes range in diameter from 2.7 mm to 4 mm, as required by the diameter of the operated ear canal. The workhorse of EES is the 4 mm 0-degree (Figure 2) due to its versatility and ease of use. The endoscope can also be equipped with an angled lens, the most common options being 30- or 45-degree lenses. These allow the surgeon to ‘view around corners’ and access areas otherwise impossible to see during MES.

### 4.1. Other Equipment

High-definition camera and monitor output: endoscopes are connected to a high-definition video camera, allowing surgeons to view the surgical field on a monitor.

Specialized instruments: miniature surgical instruments, such as micro forceps, suction devices, and lasers, are used in conjunction with the endoscope to perform various procedures.

### 4.2. Operating Room Configuration

**Figure 3:**
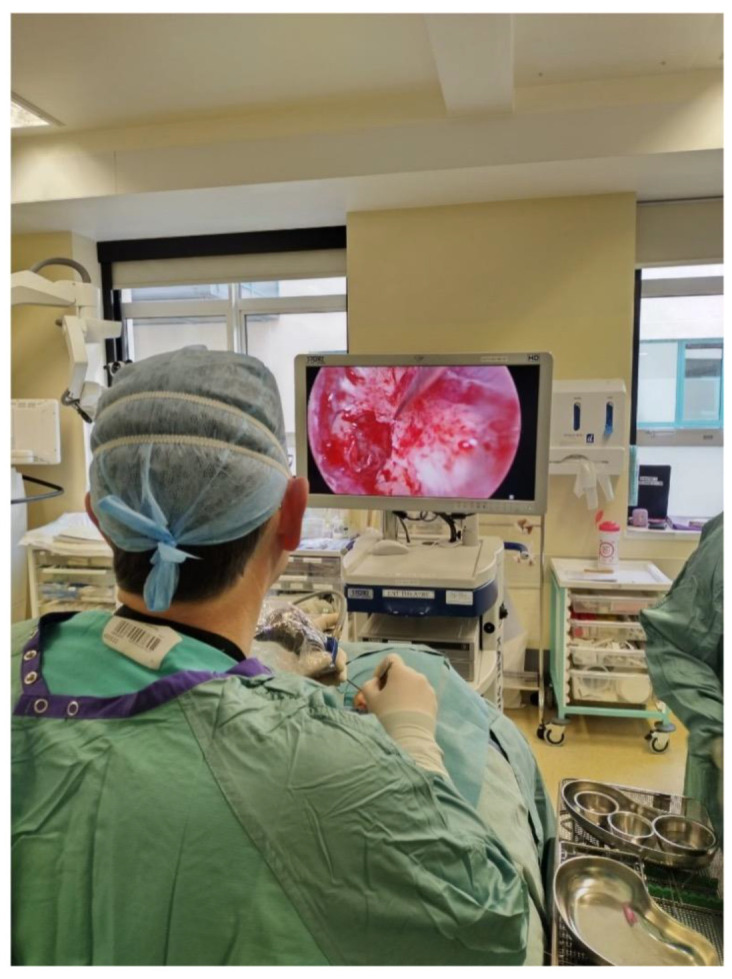
Theatre setup with a high-definition monitor positioned across the bed from the primary operator. The endoscope is held in the non-dominant (left) hand, with the camera attached, and is draped in a sterile sheath.

The typical theatre setup is shown in Figure 3. The monitor is positioned across the bed from the operating surgeon, with the monitor placed at a level that allows for ease of viewing.

## 5. Advantages of EES

### 5.1. Enhanced Visualization

As mentioned above, one of the most celebrated benefits of EES is enhanced visualization of the middle ear. The panoramic, magnified view the endoscope gives of the hidden recesses of the ear cannot be matched by MES. The areas of particular difficulty include the sinus tympani, facial recess, and eustachian tube orifice, all of which are better seen with EES. The angled endoscope also offers the ability to ‘look around corners,’ which can obviate the need for bone removal or extensive dissection that might otherwise have been necessary for clearance of disease.

### 5.2. Minimally Invasive Surgery

The ability of EES to reduce the extent of required dissection illustrates its value, even as a purely adjunctive measure. Additionally, EES can often be used to mitigate the need for external incisions, large postauricular approaches, or mastoidectomy as a means of access to the middle ear. This minimizes tissue disruption and leads to faster healing, reduced postoperative pain, and better cosmetic outcomes than would be expected with traditional ear surgery. Lastly, the minimally invasive nature of EES completely mitigates the need for head bandaging in the perioperative phase, which is particularly relevant for pediatric patients and other vulnerable groups who tolerate head bandages poorly.

### 5.3. Improved Surgical Precision

The enhanced visualization discussed above also allows for more precise surgical maneuvers, particularly when working close to critical structures like the facial nerve and bony labyrinth of the inner ear. This enhanced precision also better facilitates maximal preservation of the normal ear anatomy, which further reduces the likelihood of surgical morbidity.

### 5.4. Teaching

Otologic surgery can be challenging to teach due to the difficulty in achieving adequate visualization, as described above. These problems are further compounded during MES, as the primary surgeon is often the only one who can see the operative field in detail. The use of a camera head and digital monitor during EES enables any other member of staff to see the same images the surgeon sees and use the monitor as a point of reference for clarifying questions regarding anatomy or technique [20].

### 5.5. Ergonomics

Ergonomics plays an increasingly important role in the development of new surgical technology, and is a closely studied area in the development of new technology in ear surgery specifically [21]. EES offers clear advantages over MES, and has been proven superior in formal ergonomic analysis [22]. While both approaches provide the option of a seated operating position and both may utilize a variety of angles of approach to achieve the best view, adjusting this view in EES requires only minor adjustments of the angle of the endoscope. The monitor remains in the same position, which allows the surgeon to avoid craning their neck and back. This feature is of clear value, as neck pain has been specifically identified as a problematic issue in otorhinolaryngologists throughout their careers [23].

### 5.6. Recovery

Patients undergoing EES typically experience shorter hospital stays and faster overall recovery compared to MES [24]. The overall favourability of recovery from EES renders such patients ideal candidates for day-case surgery, a feature of vital importance in contemporary tertiary hospital services.

## 6. Limitations of EES

While endoscopic ear surgery offers numerous advantages, it is not without its challenges and limitations, which are discussed in this section.

### 6.1. One-Handed Technique

One of the main technical difficulties is the one-handed technique described above, which can limit the range of movement available and make more complex procedures difficult to perform. Indeed, regardless of complexity, there are many cases for which the need for extensive drilling or a broader exposure of the operative field can render a microscopic approach preferable. Additionally, one of the key components of surgical technique across all specialties is the use of traction and counter-traction to facilitate precise dissection. The middle ear is no exception to this, and the absence of a second hand is a limiting factor when dissecting in pressure-sensitive areas such as the stapes footplate or sinus tympani. While endoscope holders offer some remedy to this issue, the question remains as to whether patients requiring such an adjunct might be more suited to escalation to a microscopic approach [25].

### 6.2. Learning Curve

The learning curve for EES can be steep, particularly for surgeons who are more accustomed to working with MES [20]. Mastery of endoscopic techniques requires specialized training and practice to develop the necessary dexterity and hand–eye coordination, which often entails EES-focused fellowship level training. Notably, it has been shown that prior training in endoscopic sinus surgery, a key component of training in otolaryngology worldwide, is associated with a shallower learning curve in early training [26]. The lack of depth perception, a challenge inherent to two-dimensional endoscopic visualization, can further complicate procedures. This is balanced by the growing ready availability of cadaveric and virtual reality simulation models. While a recent review found that these consistently improve task performance in the simulated environment, exploration of the translation of these improvements to the clinical reality is limited [27].

### 6.3. Limited Instrumentation

Considering the evolution of EES as an adjunct to MES, it is naturally the case that many instruments employed in EES are borrowed from MES. The narrow working channels of the ear canal necessitate specially designed tools, many of which lack the flexibility and functionality required for complex cases. The need for specialized instruments has been a specific area of interest of the International Working Group on Endoscopic Ear Surgery for many years, and there is a well-described catalogue of instruments that further broadens the functionality of EES approaches. Key components of this are blends of existing instruments for both MES and endoscopic sinus surgery, including suction-assisted and hyper-angulated dissecting instruments [28]. One specific example includes the suction-assisted round knife, a modification of one of the key instruments developed specifically for MES. Nonetheless, it must be recognized that there are limits to what can reasonably be achieved with a minimally invasive approach regardless of the availability of a detailed suite of adjuncts.

### 6.4. Heat and Fogging Issues

The proximity of the endoscope’s light source to the delicate structures of the middle ear poses risks of thermal injury. While there is in vivo evidence that light-emitting diode light sources likely pose minimal risk, xenon light sources, which are commonly employed in other otolaryngology procedures, have been observed to result in temperature changes in the middle ear with prolonged use intraoperatively [29,30]. The exact significance of such temperature changes is difficult to quantify but impossible to ignore in an era where there is increasing awareness of the prevalence of intraoperative burns as a complication of surgical equipment [31]. Additionally, lens fogging, contamination of the lens with blood, and debris accumulation can all obscure visibility, necessitating frequent cleaning and interrupting surgical flow.

## 7. Applications of EES

EES is now widely used internationally, and its scope of applications has expanded significantly over the past several years. Specific procedures and the respective benefits offered by EES are listed.

### 7.1. Chronic Otitis Media and Cholesteatoma

**Figure 4:**
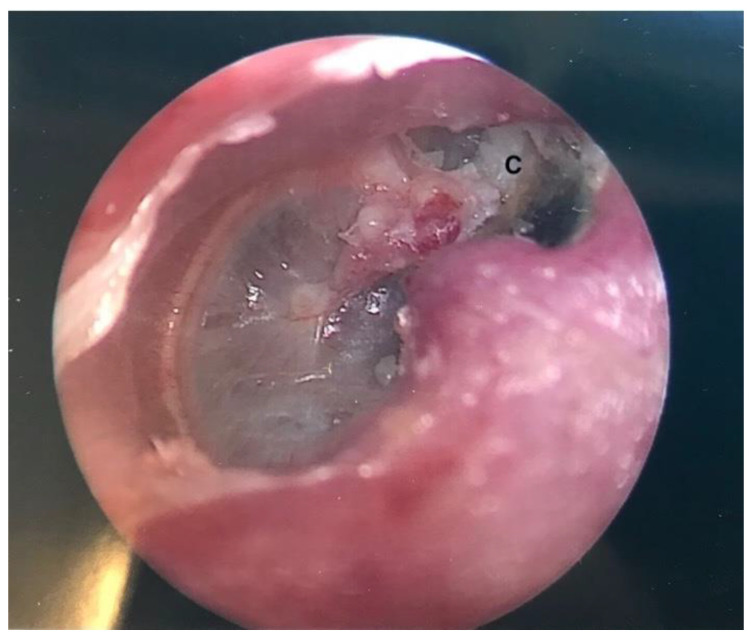
Left tympanic membrane with large cholesteatoma with auto-atticotomy. C—cholesteatoma within auto-atticotomy.

**Figure 5:**
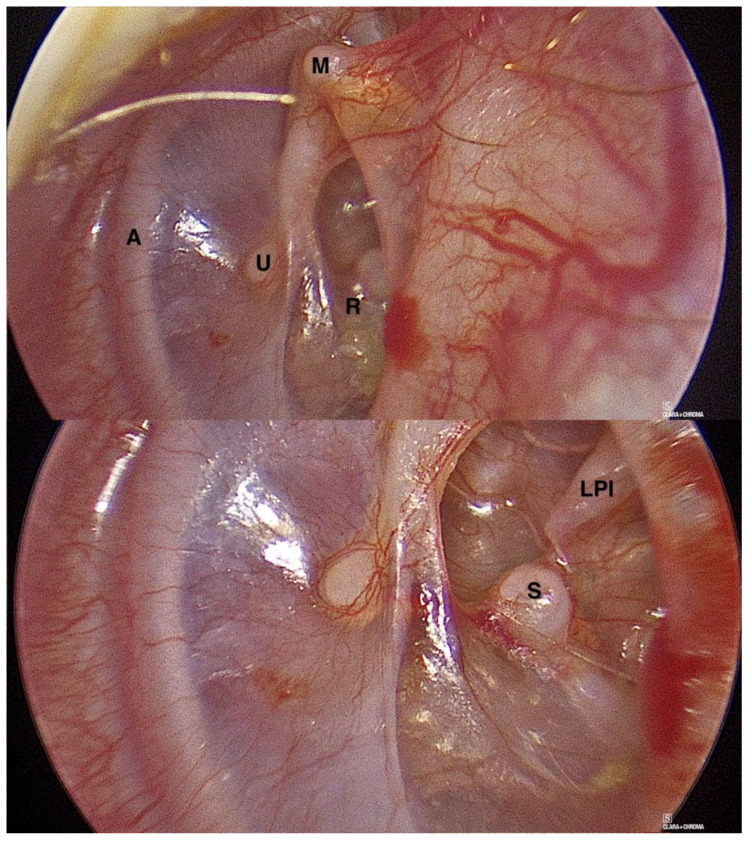
Left-sided posterior tympanic membrane retraction pocket with global view (**upper panel**) and zoomed-in view (**lower panel**) of myringostapediopexy within retraction with discontinuity of the incudostapedial joint. A—annulus; LPI—long process of incus; M—lateral process of malleus; R—retraction; S—stapes capitulum (head); U—umbo.

Chronic otitis media, particularly when associated with cholesteatoma, is a frequent indication for EES. Cholesteatoma, as shown in Figure 4, is defined by abnormal, non-cancerous deposition of keratin in the middle ear. EES is helpful in the diagnostic phase of cholesteatoma for the detailed examination of retraction pockets (Figure 5), which may contain cholesteatoma. EES also enables the precise removal of cholesteatoma in the therapeutic phase, particularly in difficult-to-reach areas, as discussed above. The use of angled endoscopes ensures complete removal, reducing the risk of recurrence and the requirement for revision surgery. Despite the relatively minimally invasive nature of EES, the prevalence of recurrence appears comparable or even superior to MES with the added benefits of reduced post-operative pain and vertigo [32,33].

### 7.2. Tympanoplasty

**Figure 6:**
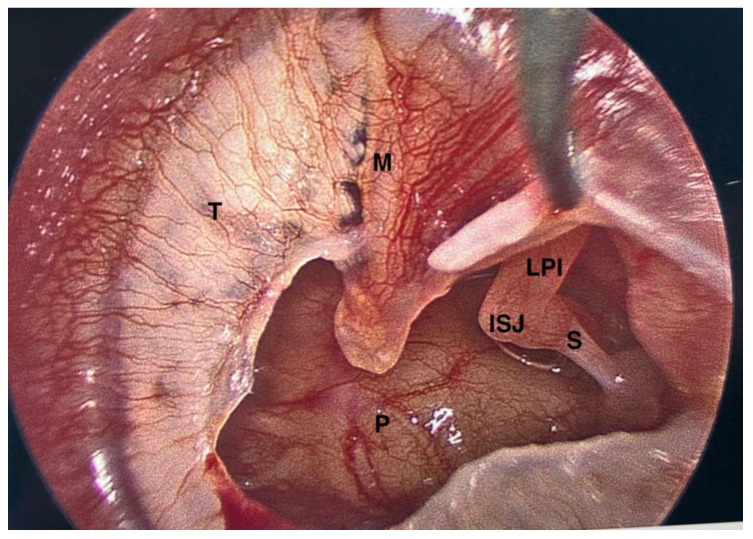
Large posteroinferior left tympanic membrane perforation with exposure of the incudostapedial joint and stapes superstructure. ISJ—incudostapedial joint; LPI—long process of incus; M—handle of malleus; P—cochlear promontory; S—stapes; T—tympanic membrane with tympanosclerosis.

Tympanoplasty, or the surgical repair of tympanic membrane perforations (Figure 6), is another standard procedure performed endoscopically. The enhanced visualization improves the precision of graft placement, while the minimally invasive nature of the approach eliminates the need for a postauricular incision. There is high-quality evidence that EES in tympanoplasty results in decreased operative time, reduced post-operative pain, and lower complication rates when compared with MES [34,35,36]. One recent systematic review goes further again and concludes that EES should be the procedure of choice unless contraindicated [37].

### 7.3. Ossiculoplasty

Ossiculoplasty is the reconstruction of the ossicular chain to restore hearing in patients with conductive hearing loss. EES provides a direct view of the ossicular chain, facilitating precise placement of prosthetic devices or ossicular reconstruction materials. This reduces the risk of morbidity and damage to surrounding structures, decreases postoperative pain, and yields shorter operative time with comparable audiologic outcomes to MES [38,39].

### 7.4. Stapes Surgery

Endoscopic techniques are also used in stapedectomy or stapedotomy. These procedures are designed to treat otosclerosis, a condition in which the stapes bone becomes fixed, resulting in hearing loss. Endoscopic visualization enables more accurate dissection around the stapes footplate and more precise placement of prosthetic devices. Again, endoscopic stapes surgery has been shown to be equivalent to microscopic stapes surgery in terms of audiologic outcomes and superior across a broad range of patient-reported and surgical outcomes, including postoperative dysgeusia, pain, and visualization of the incudo-stapedial joint [40,41].

### 7.5. Cochlear Implantation

Building on the success of EES in the middle ear, multiple centres have now described endoscopic-assisted cochlear implantation [42,43,44,45]. The literature on this topic primarily discusses this approach as indicated in any setting where there is inadequate exposure of the round window niche. Specifically, this has proven useful where there is aberrance of the course of the facial nerve. EES for transcanal cochlear implantation is also described as an alternative approach which obviates the need for mastoidectomy by placing the electrode array in a drilled trough that follows the course of the external auditory canal [46]. The quality of exposure offered by EES has also led to the identification of landmarks, such as the fustis, a bony ridge on the floor of the round window region, which serves as a constant landmark for the scala tympani [47]. There is a need for more research into patient-reported outcomes in this area.

### 7.6. Skull Base Surgery

A growing body of literature now exists on EES as an adjunct and as a means of accessing the lateral skull base. This work is built upon the extensive foundation of endoscopic access to the anterior skull base, for which a variety of endonasal and transorbital approaches are described [48]. Transcanal access to the skull base has now been described for a variety of pathologies, including cholesteatoma with petrous temporal bone or inner ear involvement, vestibular schwannoma, facial nerve haemangioma, and meningioma [49]. Three approaches have been described based on the region of the skull base that is the target of the planned procedure: the suprageniculate corridor for the interface between the labyrinth and the middle cranial fossa, the transpromontorial corridor for the internal auditory canal and cerebellopontine angle, and the infracochlear corridor for the inferior portion of the petrous apex. EES would clearly reduce the need for extensive bony dissection in patients with suitable disease anatomy, but in this setting again is more commonly reported as an adjunct [50]. There is little reported data on the use of TEES for lateral skull base surgery, though this continues to improve in recent years.

## 8. Future Directions

The field of endoscopic ear surgery is rapidly evolving, and several promising developments and adjuncts are on the horizon.

### 8.1. Training

Surgical training has undergone continuous evolution over the past several hundred years. Surgical training was historically undertaken in accordance with Halstedian principles, an approach that has stood the test of time remarkably well. Nonetheless, given the availability of cadaveric material and the complexity of interventions now possible, it is no longer acceptable that a trainee’s first foray into complex ear surgery be on a live patient. In this vein, the future of EES training will likely merge classic cadaveric model prosections with the development of virtual reality platforms featuring detailed anatomy and interactive capabilities. Some procedure-specific (myringotomy and cochlear implantation) platforms have been developed, although none yet exist for EES [51].

### 8.2. Instrumentation

As highlighted above, one of the current limitations of EES is the ad hoc integration of instruments that are not purpose-designed. There is a proud tradition of surgeon-led development of surgical instruments in otology, many of which were first manufactured in the 19th and 20th centuries [52]. There is a need for the development of EES-specific instruments and adjuncts to address common intraoperative pitfalls. For example, in endoscopic sinus surgery, continuous lens irrigation systems are in wide circulation to address the above highlighted issues with lens fogging and blood straining. These range from sophisticated pump-powered devices to more pragmatic systems that utilize readily available equipment in any hospital system [53]. While such systems can be applied to EES, this area remains less well explored. The volume of irrigation employed in sinus surgery systems is also counterproductive in the confined space of the middle ear. Despite this, recent experiences with continuous irrigation during EES have been described and show significant promise [54].

### 8.3. Imaging Technology in Ear Surgery

Advances in imaging technology, such as 3D endoscopy, are likely to enhance the surgeon’s ability to visualize and operate on the middle ear in even greater detail. Multiple 3D exoscopic systems are now commercially available and have been described as combining the relative advantages of microscopic surgery (two-handed technique) and EES (image detail and improved ergonomics) [55,56,57,58]. It is noted that this technology is still in its infancy for ear surgery and does not yet represent a viable substitute for the microscope or endoscope. However, this may change as the technology develops. There is also a rapidly developing field of intraoperative imaging enhancement, which shows promise both for enhancing available images and utilizing artificial intelligence as a decision-making aid [59,60,61]. Finally, image-guided navigation systems have become nearly ubiquitous in anterior skull base surgery and neurosurgery, but clinical use in lateral skull base surgery is still limited to small series and case reports. There are multiple reasons for this, including the low tolerance for registration error in the tight confines of the middle ear and the limited number of cases for which this modality might offer significant improvements in outcome [62]. Nonetheless, cadaveric data seems to indicate that acceptable registration error may be achievable with currently available systems, which may in time translate to clinically useful applications [63].

### 8.4. Robotic-Assisted Surgery

Robotics holds significant potential for the future of EES. Robotic-assisted surgery could overcome some of the technical challenges associated with single-handed endoscopy by providing precision and control not possible within the limits of human dexterity. The primary targets of development in this area include more accurate stapes surgery, minimally invasive access to the cochlea, and less traumatic insertion of cochlear implant electrode arrays [64]. In 2024, the first instance of totally robotic cochlear implantation via the HEARO platform was reported by Abari et al.; this represents the state of the art in cochlear implant surgery, but it is unclear how practically feasible this approach will be in the near future [65]. Similarly, the RobOtol platform was used in a case series for a range of middle ear procedures with excellent surgical results, though no information was provided on patient-reported outcomes in this cohort [66]. Ultimately, cost and regulatory barriers are likely to delay routine use of these platforms for many years to come [67].

### 8.5. Research

Lastly, an issue that has historically faced EES is the relative lack of high-quality randomized controlled trials proving its utility and safeguarding its future. With the publication of the above referenced systematic review and meta-analyses and, as more granular retrospective outcome data becomes available, the demonstrably lower morbidity of EES becomes more widely apparent. In recent years, randomized controlled trials are increasingly focusing on surgical outcomes, including operative time, recurrence, and surgical complications such as chorda tympani injury, but also on patient-reported outcomes, including quality of life, pain, vertigo, and hearing [68,69,70,71]. It is encouraging that patients who undergo a minimally invasive approach seem to have less pain, vertigo, and chorda tympani injury, as one would expect if the principles underpinning EES translate to clinical reality. This can only serve to cement EES as part of standard otologic training and practice, but more work in this area is needed.

## 9. Conclusions

EES represents a significant advancement in the field of otology, offering a less invasive, more precise alternative to traditional microscopic techniques. With its ability to enhance visualization, minimize tissue disruption, and improve patient outcomes, EES has become an invaluable tool for treating a variety of ear disorders. As technology continues to advance, the role of endoscopic techniques in ear surgery is likely to expand further, bringing about new possibilities for minimally invasive procedures and improved patient care.

## Data Availability

No new data were created or analyzed in this study.

## Abbreviations

The following abbreviations are used in this manuscript:

EES	endoscopic ear surgery
MES	microscopic ear surgery
TEES	totally endoscopic ear surgery
